# Calcified Constrictive Pericarditis (Concretio Cordis) in a Renal Transplant Recipient: A Multimodal, Hemodynamic, and Surgical Challenge

**DOI:** 10.7759/cureus.114121

**Published:** 2026-08-07

**Authors:** Yandri F Quiroz, Marco V Zapata, Marco Zapata, Julio C Paredes, Mauricio Peralta

**Affiliations:** 1 Cardiology, Pontificia Universidad Católica del Ecuador, Quito, ECU; 2 Cardiothoracic Surgery, Hospital de Especialidades Carlos Andrade Marín, Quito, ECU; 3 Cardiology, Hospital de Especialidades Carlos Andrade Marín, Quito, ECU

**Keywords:** echocardiography doppler, immunosuppression, kidney transplantation, pericardiectomy, pericarditis constrictive

## Abstract

Calcified constrictive pericarditis is an uncommon but severe clinical entity that causes restrictive right-sided heart failure. Its occurrence in renal transplant recipients presents a highly complex scenario due to interactions with immunosuppression, the risk of graft dysfunction caused by hemodynamic instability, and the surgical challenges of decortication. We report a 45-year-old male, renal transplant recipient (2018) on maintenance therapy with tacrolimus, everolimus, and prednisone, who was evaluated for progressive refractory ascites that masked the underlying cardiovascular disease. Computed tomography and transthoracic echocardiography revealed massive 6 mm pericardial thickening with complete circumferential calcification (concretio cordis) and pathognomonic hemodynamic signs: early diastolic septal bounce, a 30% respiratory variation in transmitral flow, and annulus reversus (septal E' greater than lateral E'), associated with secondary congestive hepatopathy and moderate thrombocytopenia. He underwent subtotal off-pump pericardiectomy via median sternotomy. During decortication of the 0.5 cm thick calcified pericardium, incidental injuries occurred in the right atrium and the right ventricular outflow tract, which were successfully repaired using bovine pericardial patches and autotransfusion with a Cell Saver system. The postoperative course showed hemodynamic stability, preservation of graft function, and a drastic weight reduction (from 51.2 kg to 49 kg) due to ascites volume depletion. During mid-term outpatient follow-up, the patient achieved complete remission to New York Heart Association (NYHA) functional class I, with definitive resolution of the ascitic syndrome and normalization of electrocardiographic and functional echocardiographic parameters, highlighting the disappearance of septal bounce and restoration of inferior vena cava collapse greater than 50%. In conclusion, pericardial constriction is a clinically elusive condition that mimics primary liver disease, meaning it must be included in the differential diagnosis of refractory ascites in transplant patients. Therapeutic success relies on diagnostic suspicion, transdisciplinary integration, and safe surgical decortication to preserve the allograft.

## Introduction

Calcified constrictive pericarditis (CCP), also known historically as concretio cordis, represents the most extreme and irreversible spectrum of chronic pericardial inflammation, characterized by extensive, dense calcium deposition within a fibrotic, thickened pericardial sac [1,2]. This rigid encasement progressively obliterates the pericardial space, restricting normal diastolic expansion and ventricular filling, leading to dissociation of intrathoracic and intracardiac pressures, severe systemic venous congestion, and ultimately, intractable right-sided heart failure [3].

Although the incidence of CCP in developed nations has significantly declined, largely attributed to the near-eradication of tuberculous pericarditis, it remains a formidable clinical challenge with diverse etiologies, including prior cardiac surgery, radiation therapy, and chronic uremia [4]. In the highly specific subset of patients with a history of solid organ transplantation, particularly renal transplant recipients, CCP is an exceptionally rare phenomenon [5]. This population is uniquely vulnerable due to complex pathophysiological interactions, including long-standing pre-transplant chronic kidney disease, previous prolonged hemodialysis, potential subclinical opportunistic infections secondary to immunosuppression, and chronic systemic inflammation [6].

The diagnostic approach in this demographic is often complicated by an insidious and delayed clinical presentation, where classic symptoms of heart failure are frequently masked or misinterpreted as chronic hepatopathy or graft-related fluid overload [7]. Consequently, patients frequently endure a prolonged course of profound systemic congestion, marked by anasarca, refractory ascites, and cachexia, before a definitive diagnosis is established.

The optimal management of CCP is exclusively surgical, necessitating an extensive pericardiectomy [8]. However, in renal transplant recipients, surgical intervention carries exorbitant morbidity and mortality risks. The conventional use of cardiopulmonary bypass (CPB) during pericardiectomy exacerbates these risks, precipitating severe systemic inflammatory response syndrome (SIRS), profound coagulopathy, and, critically, acute kidney injury (AKI) that threatens the survival of the solitary functioning allograft [8,9]. To mitigate these profound complications, an off-pump surgical strategy has emerged as a promising alternative, aiming to maximize allograft protection while achieving complete hemodynamic relief [9].

When compared with previously published reports of renal transplant recipients undergoing pericardiectomy for constrictive pericarditis, our case shares similarities regarding the long-standing pre-transplant uremic background but stands out due to the extreme severity of massive pericardial calcification (concretio cordis) completely mimicking advanced liver disease [8,9]. Furthermore, while historical or alternative approaches frequently relied on CPB, our successful application of an off-pump surgical strategy underscores a critical technical distinction aimed at prioritizing solitary renal allograft protection and achieving favorable postoperative recovery [8,9].

We present a rare and highly instructive case of severe concretio cordis mimicking advanced chronic liver disease in a functioning renal transplant recipient, successfully managed through an advanced multimodal diagnostic pathway and definitively treated with a protective subtotal off-pump pericardiectomy.

## Case presentation

A 52-year-old male with a history of end-stage renal disease of undetermined etiology, having been on chronic hemodialysis for several years. In 2018, he received a deceased donor renal transplant located in the right iliac fossa. The evolution of the graft was complicated by BK virus nephropathy, managed with a reduction in immunosuppression and the use of immunoglobulins, followed by acute T-cell-mediated rejection IB, effectively treated with anti-thymocyte globulin (thymoglobulin) and systemic corticosteroids, resulting in stabilization of baseline renal function (serum creatinine 1.5-1.7 mg/dL). His maintenance immunosuppressive regimen consisted of everolimus 1.5 mg every 12 hours, mycophenolate sodium 360 mg every 12 hours, and prednisone 5 mg daily.

The patient was admitted due to refractory ascites of unknown etiology, with a history of approximately 18 months, requiring two large-volume therapeutic paracenteses and optimal medical therapy with oral loop diuretics (furosemide 40 mg every 12 hours) and dietary sodium restriction. Upon admission, he was cardiovascularly asymptomatic at rest, without marked exertional dyspnea, orthopnea, or chest pain. Initial physical examination revealed vital signs within normal limits (blood pressure 110/70 mmHg, heart rate 78 bpm, respiratory rate 16 breaths/min, oxygen saturation 96% on ambient air). Relevant clinical findings included marked bilateral lower extremity edema (3+/4+), prominent jugular venous distention, congestive hepatomegaly without stigmata of chronic liver disease (spider angiomas, palmar erythema), and neurological status with a Glasgow Coma Scale score of 15/15. The initial hemodynamic profile was classified as American Heart Association (AHA)/American College of Cardiology (ACC) Stage C, New York Heart Association (NYHA) Class II.

Routine laboratory investigations were performed upon admission. Baseline results revealed mild azotemia within a stable renal allograft baseline, a marked cholestatic pattern, and secondary hypersplenism driven by chronic passive venous congestion. This was further accompanied by a high serum-ascites albumin gradient (SAAG) (> 1.1 g/dL) in the peritoneal fluid analysis. The complete preoperative laboratory profile and biochemical markers of the patient are detailed in Table 1.

**Table 1:** Preoperative analytical summary of blood biomarkers

Laboratory Parameter	Obtained Value	Reference Range	Status/Interpretation
Total Leukocytes (WBC)	5.05 × 10³/µL	4.50-11.00 × 10³/µL	Normal
Hemoglobin (HGB)	12.4 g/dL	13.0-17.0 g/dL	Decreased (mild anemia)
Platelet Count	87,000 /µL	150,000-450,000 /µL	Decreased (thrombocytopenia)
Serum Creatinine	1.31 mg/dL	0.70-1.30 mg/dL	Elevated (mild renal dysfunction)
Serum Urea	71.1 mg/dL	15.0-40.0 mg/dL	Elevated (uremic azotemia)
Blood Urea Nitrogen (BUN)	33.0 mg/dL	8.0-20.0 mg/dL	Elevated
Gamma-Glutamyl Transferase (GGT)	356 U/L	< 60.0 U/L	Elevated (marked cholestatic pattern)
Alkaline Phosphatase (ALP)	171 U/L	< 120.0 U/L	Elevated
Total Bilirubin	0.712 mg/dL	0.200-1.200 mg/dL	Normal
Direct Bilirubin	0.366 mg/dL	0.000-0.300 mg/dL	Elevated (mild hyperbilirubinemia)
Aspartate Aminotransferase (AST)	28.9 U/L	< 40.0 U/L	Normal
Prothrombin Time (PT)	12.3 seconds	11.0-13.5 seconds	Normal
International Normalized Ratio (INR)	1.12	0.85-1.20	Normal
Serum Sodium (Na+)	138.5 mEq/L	135.0-145.0 mEq/L	Normal
Serum Potassium (K+)	4.01 mEq/L	3.50-5.00 mEq/L	Normal

**Figure 1:**
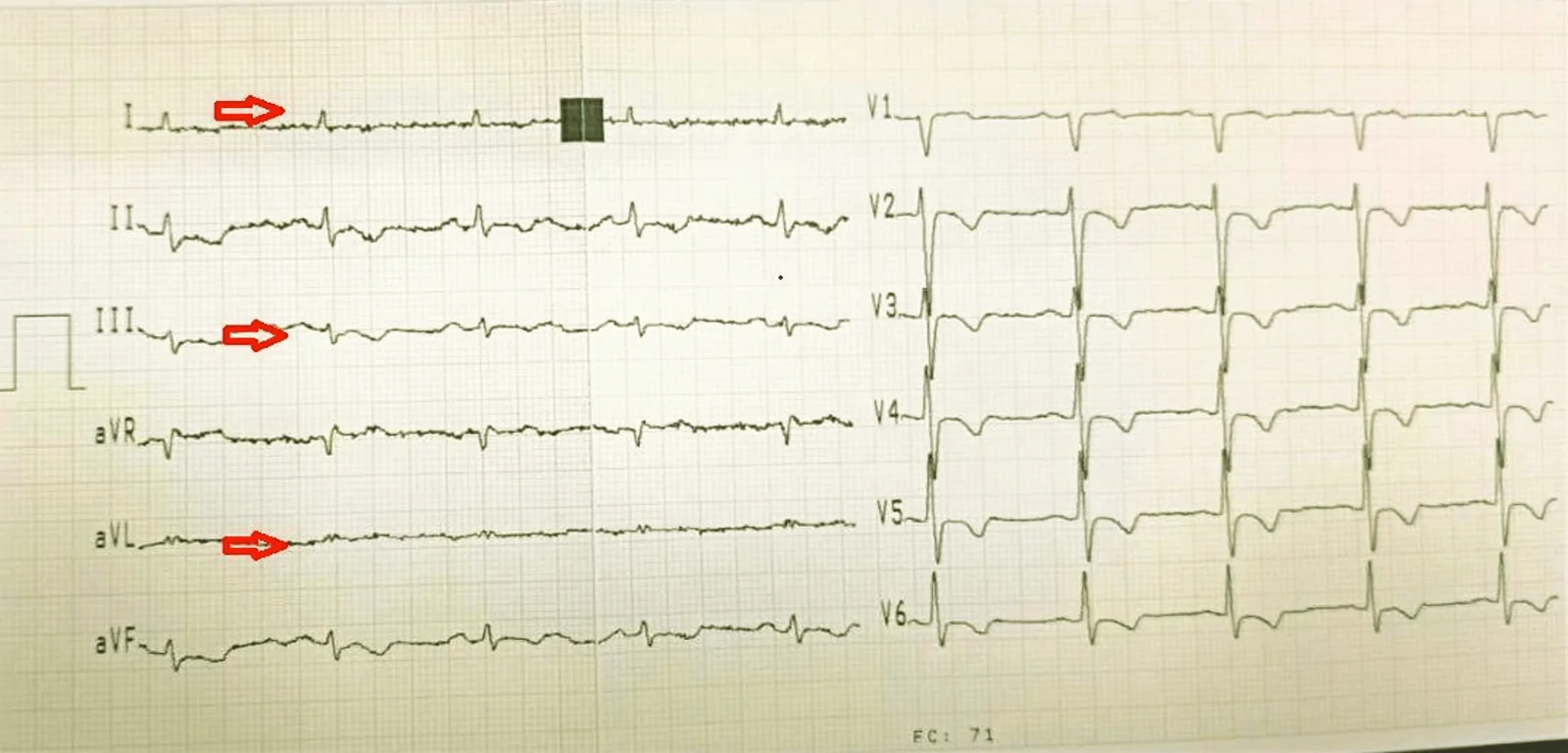
The 12-lead electrocardiogram Demonstrates sinus rhythm at 78 bpm with generalized low voltage in the limb leads, signs of left atrial enlargement (P mitrale), and nonspecific ventricular repolarization abnormalities with negative T waves from V2 to V6 and in the inferior leads, findings consistently described in pericardial restriction. The red arrows indicate the generalized low voltage (microvoltage) in the limb leads.

The 12-lead electrocardiogram showed sinus rhythm at 78 bpm with generalized low voltage in the limb leads, signs of left atrial enlargement (P mitrale), and nonspecific ventricular repolarization abnormalities with negative T waves from V2 to V6 and in the inferior leads, findings consistently described in pericardial restriction (Figure 1).

**Figure 2:**
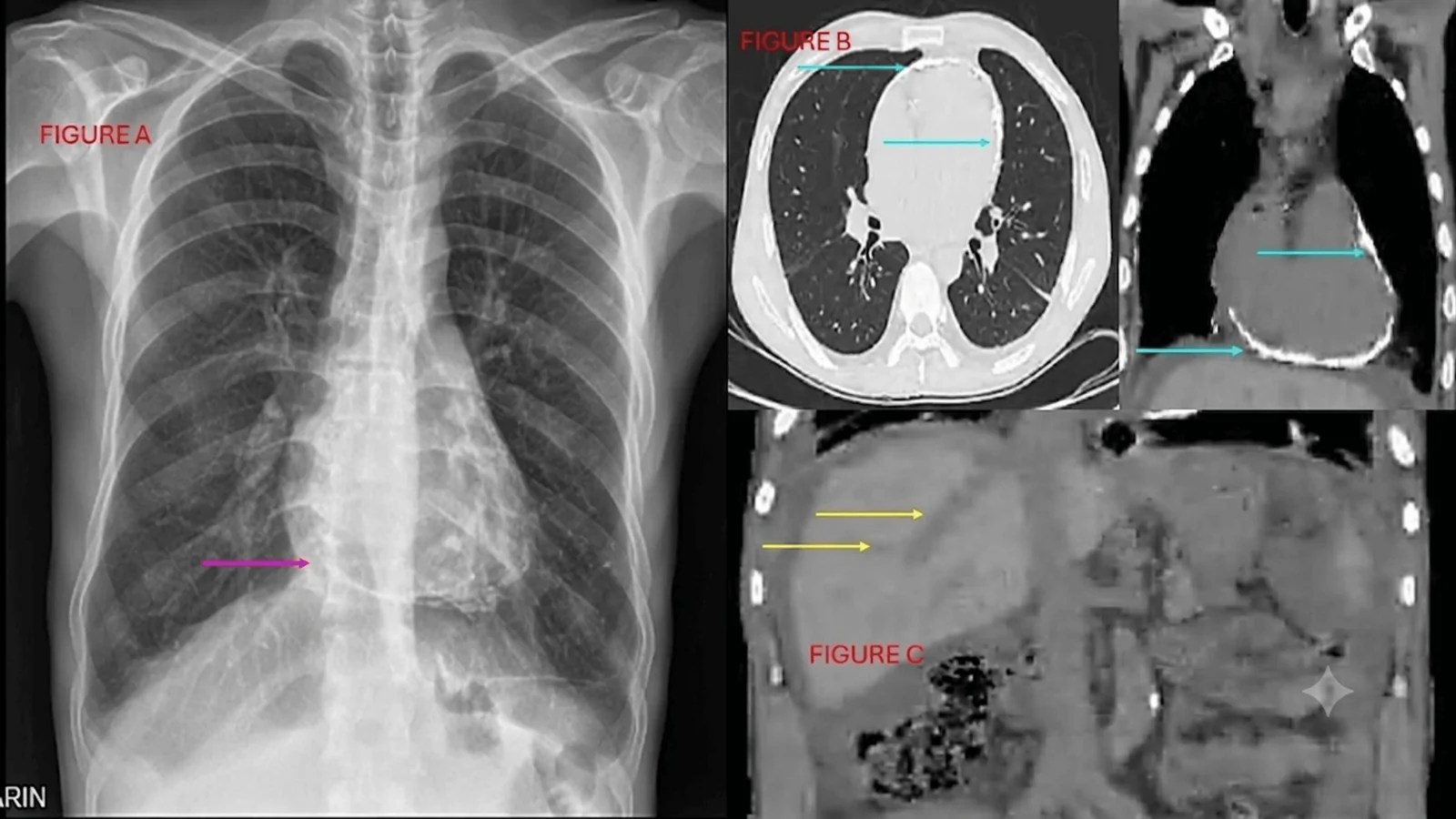
Multimodal diagnostic evaluation using thoracoabdominal imaging (A) Chest X-ray (posteroanterior (PA) view): The magenta arrow indicates a thin peripheral radiopaque line of pericardial calcification. (B) Chest CT (axial view): The cyan arrows show massive, circumferential pericardial calcification ("armor heart"). (C) Abdominal CT: The yellow arrows point to the marked dilation of the hepatic veins due to high pressure.

High-resolution chest computed tomography (CT) confirmed the anatomical diagnosis of concretio cordis, demonstrating massive, circumferential calcification of the pericardium surrounding all cardiac chambers ("armor heart"), with a measured thickness of 6 mm and the absence of significant free effusion (Figure 2).

Abdominal ultrasound and CT angiography showed ascites quantified at 1153 cc, congestive hepatomegaly with dilated hepatic veins, persistence of a left iliac deep vein thrombosis (DVT), and a transplanted kidney located in the right iliac fossa with preserved morphology.

**Figure 3:**
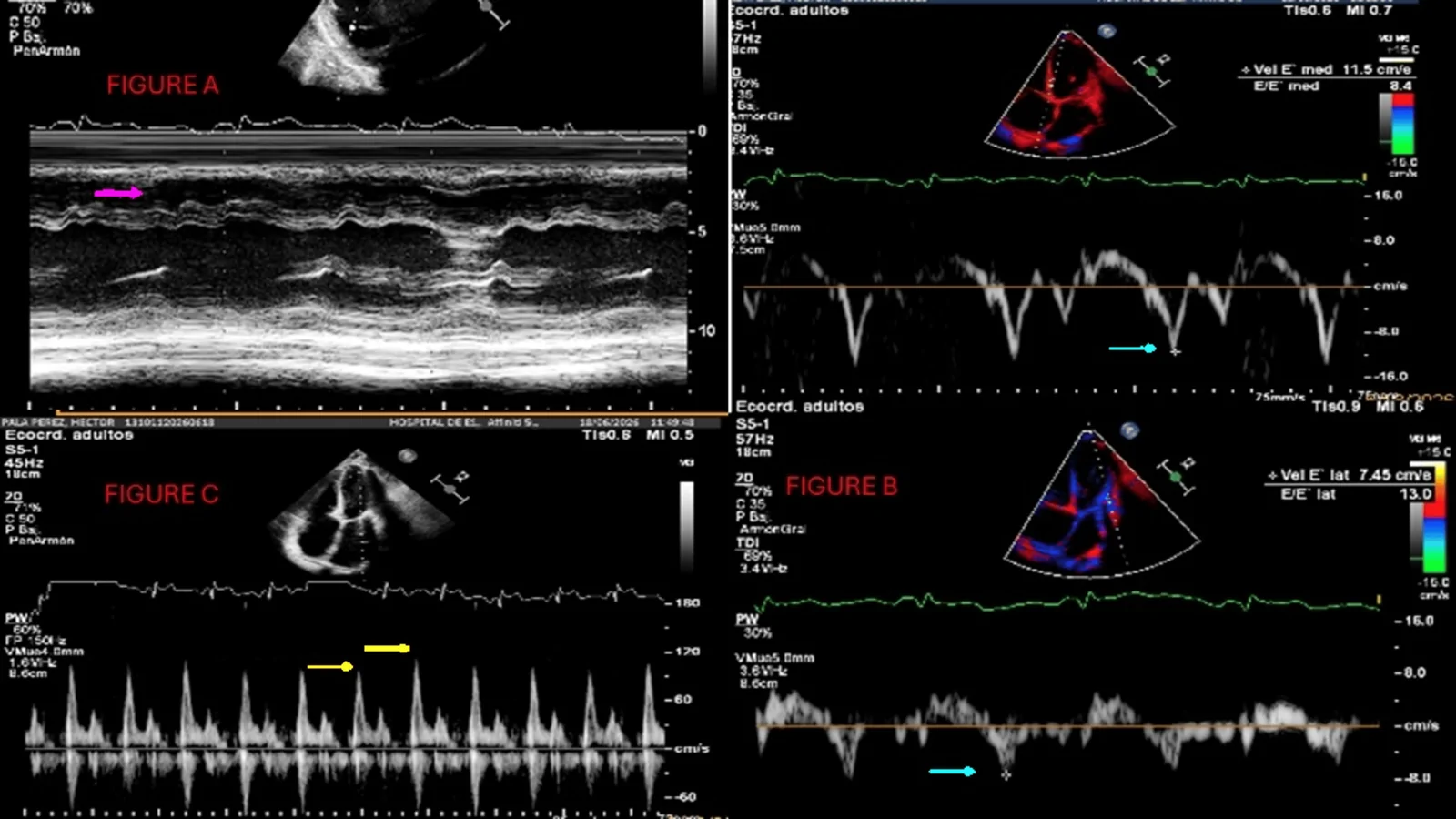
Pathognomonic functional echocardiographic and tissue Doppler findings (A) Echocardiogram (M-mode): The magenta arrow indicates the abrupt early diastolic septal bounce. (B) Tissue Doppler: The cyan arrows confirm annulus reversus (septal E' velocity paradoxically higher than lateral E'). (C) Transmitral Doppler: The yellow arrows show the 30% respiratory variation in E-wave velocity.

The quantitative Doppler transthoracic echocardiogram provided definitive confirmation of the constrictive physiology, ratifying the Mayo Clinic criteria for constrictive pericarditis (Figure 3).

Regarding ventricular dimensions and function, a preserved left ventricular ejection fraction (LVEF) of 55% was observed using Simpson's method, with a septal and posterior wall thickness of 0.8 cm, a left atrial diameter of 4.0 cm, and depressed right ventricular systolic function with a tricuspid annular plane systolic excursion (TAPSE) of 13 mm. Extreme ventricular interdependence was evidenced by an abrupt early diastolic septal bounce. Furthermore, a dissociation of intrathoracic pressures was observed, with a 30% respiratory variation in transmitral flow (apical E wave of 96.8 cm/s, E/A ratio of 2.2, and deceleration time of 130 ms), compatible with a grade III restrictive diastolic dysfunction. Tissue Doppler imaging (TDI) documented the annulus reversus phenomenon, characterized by an inversion of mitral annular velocities, with a septal E' velocity (11.5 cm/s) paradoxically higher than the lateral E' velocity (7.45 cm/s). Finally, the assessment of filling pressures revealed marked biatrial dilation (indexed left atrial volume of 42.2 mL/m² and right atrial volume of 21 mL/m²), along with a dilated inferior vena cava of 2.7 cm without inspiratory collapse, allowing an estimated right atrial pressure (RAP) of 20 mmHg and a pulmonary artery systolic pressure (PASP) of 45 mmHg.

**Figure 4:**
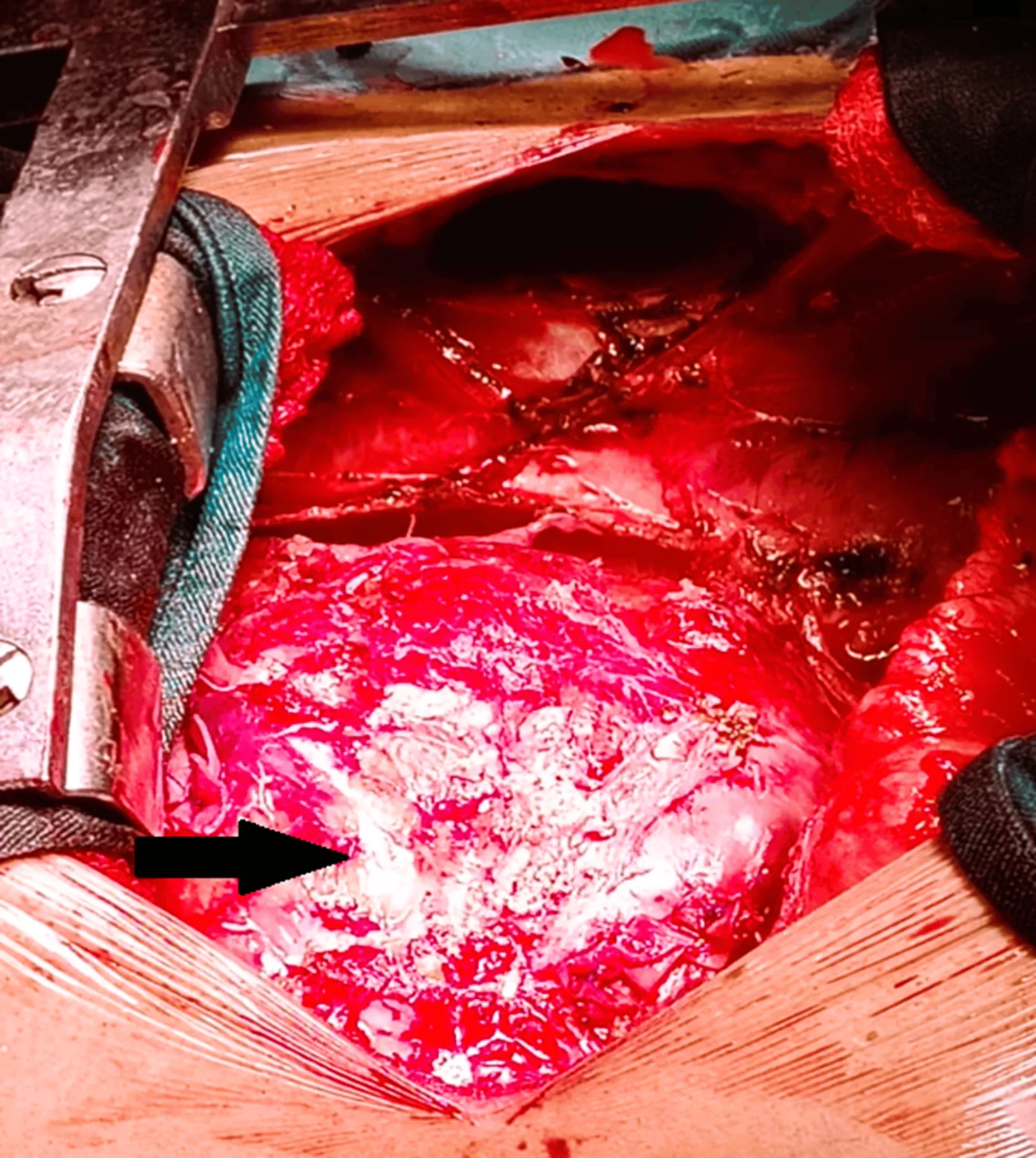
Initial macroscopic operative field Macroscopic operative field. The black arrow indicates the severely thickened pericardial sac and dystrophic calcification plaques before resection.

The patient was scheduled for a definitive surgical intervention on June 26, 2026. The perioperative plan included creatinine monitoring to watch graft function, strict fluid balance control, hemostasis management due to thrombocytopenia, medication reconciliation via inpatient bridging anticoagulation with enoxaparin, and the maintenance of immunosuppression with tacrolimus and prednisone. Under balanced general anesthesia, a midline median sternotomy of approximately 25 cm was performed, achieving exposure of the anterior mediastinum, the pericardial sac, and the great vessels (Figure 4).

Severe pericardial involvement was identified, with a fibrous thickening and massive circumferential dystrophic calcification of approximately 0.5 cm in thickness that confined the entire myocardium, causing a global extrinsic cardiac constriction.

A subtotal off-pump pericardiectomy was performed without CPB support, extending anatomically from phrenic nerve to phrenic nerve, including complete liberation of the diaphragmatic surface. This off-pump strategy was explicitly selected to avoid CPB-induced systemic inflammation, coagulopathy, and renal hypoperfusion, thereby maximizing the protection of the renal allograft (Video 1). 

**Video 1:** Initiation of subtotal off-pump pericardiectomy Dynamic intraoperative recording showing the initial incision and the first dissection planes over the calcified anterior pericardium, evidencing the contractile movement of the myocardium restricted by the rigid armor.

During the active decortication phase over the densely adherent and calcified tissue, two intraoperative complications occurred: an incidental injury to the right atrium and another in the right ventricular outflow tract (Video 2). 

**Video 2:** Decortication and myocardial liberation Dynamic recording of blunt dissection and resection of the calcified pericardial block (thickness ~0.5 cm). The meticulous surgical maneuver required to detach the calcium plaques strongly adhered to the epicardium of the right ventricle is documented.

Both tissue disruptions were successfully resolved by direct suture with 4-0 and 5-0 polypropylene (Prolene), respectively, anchored to bovine pericardial patches (Video 3). 

**Video 3:** Resection of the surgical specimen for histopathological examination Intraoperative dynamic recording illustrating the excision and removal of the major section of the markedly thickened and calcified pericardium (pericardial shell), destined for definitive histopathological analysis for etiological confirmation of the disease.

Total estimated blood loss was 1000 cc, mitigated by an intraoperative blood salvage system (Cell Saver), which allowed autotransfusion of a volume of 900 mL. Following resection, an immediate optimization of cardiac compliance was verified. A 32 Fr mediastinal drainage tube and a Jackson-Pratt closed suction system were placed.

In the postoperative period, the patient showed a favorable clinical course and immediate resolution of the constrictive physiology. Vital signs demonstrated persistent hemodynamic stability without vasopressor support (heart rate: 93 bpm; blood pressure: 106/69 mmHg; oxygen saturation: 93% on room air with a FiO₂ of 21%). A critical clinical finding was a drastic reduction in body weight, decreasing from the pre-intervention baseline weight to a recorded weight of 49 kg, with an intermediate control of 51.2 kg. This massive decrease reflected the depletion of volume accumulated by the refractory ascites and generalized edema after systemic hydrostatic pressures normalized. Renal graft function was closely monitored throughout the intraoperative and immediate postoperative period, remaining stable and free of dysfunction. Postoperative radiographic controls confirmed the removal of the armor and adequate lung expansion. 

Resected pericardial tissue samples were sent for definitive histopathological study to determine the underlying etiology of the calcified fibrosis.

**Figure 5:**
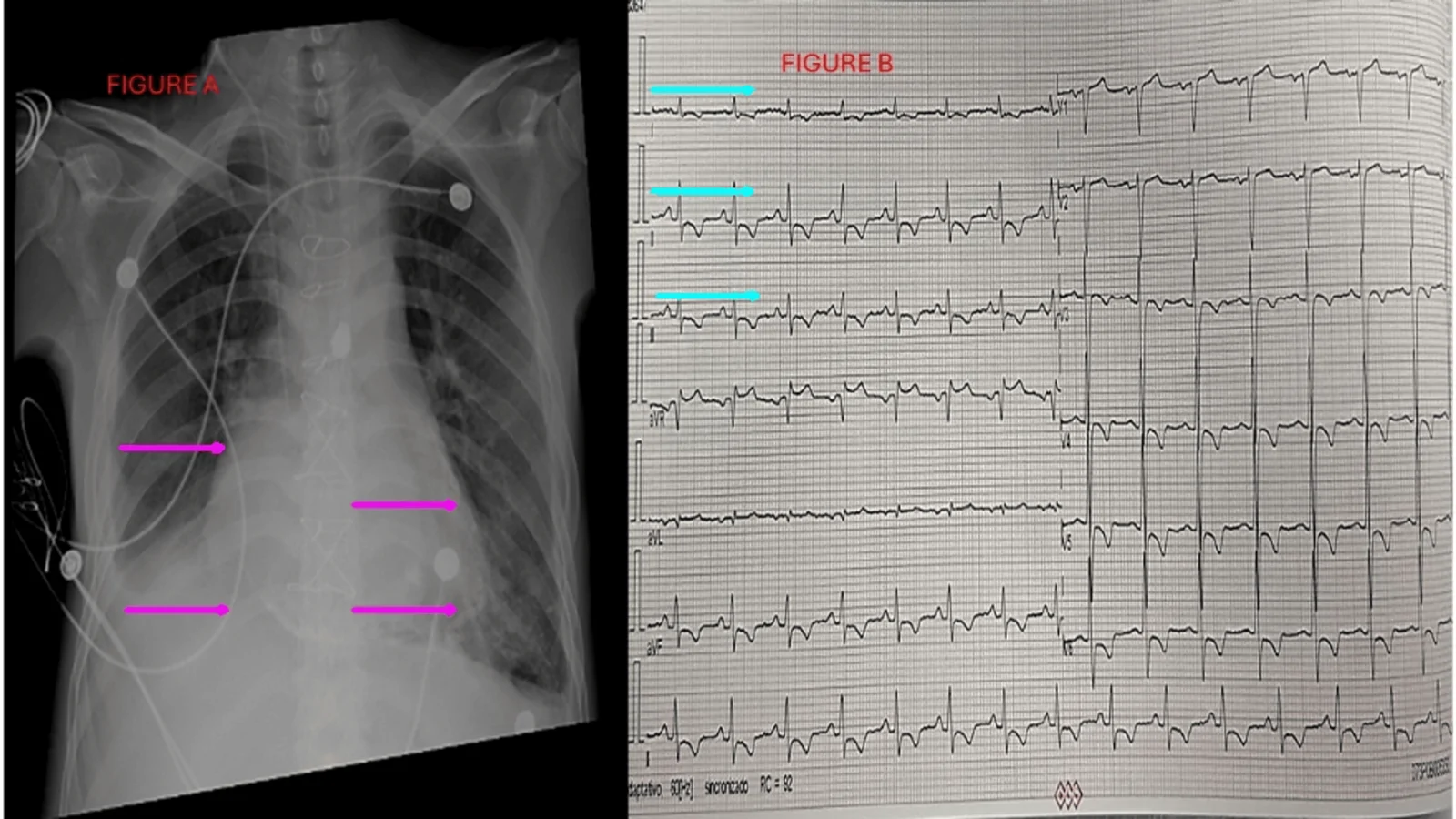
Evolutionary postoperative radiographic and electrocardiographic evaluation (A) Chest X-ray (anteroposterior (AP) view): The magenta arrows show adequate bilateral lung expansion and complete removal of the pericardial radiopaque density. (B) Follow-up electrocardiogram: The cyan arrows indicate the significant increase in QRS complex voltage amplitude following myocardial release.

During mid-term outpatient follow-up, the patient achieved complete clinical recovery, remaining in NYHA functional class I, with definitive resolution of the ascitic syndrome and lower extremity edema, allowing a return to regular activities. Paraclinical control studies demonstrated an evolutionary normalization in multimodal imaging methods. The outpatient electrocardiogram showed a significant recovery of QRS complex voltages in the limb leads and attenuation of the previously described ventricular repolarization abnormalities (Figure 5).

**Figure 6:**
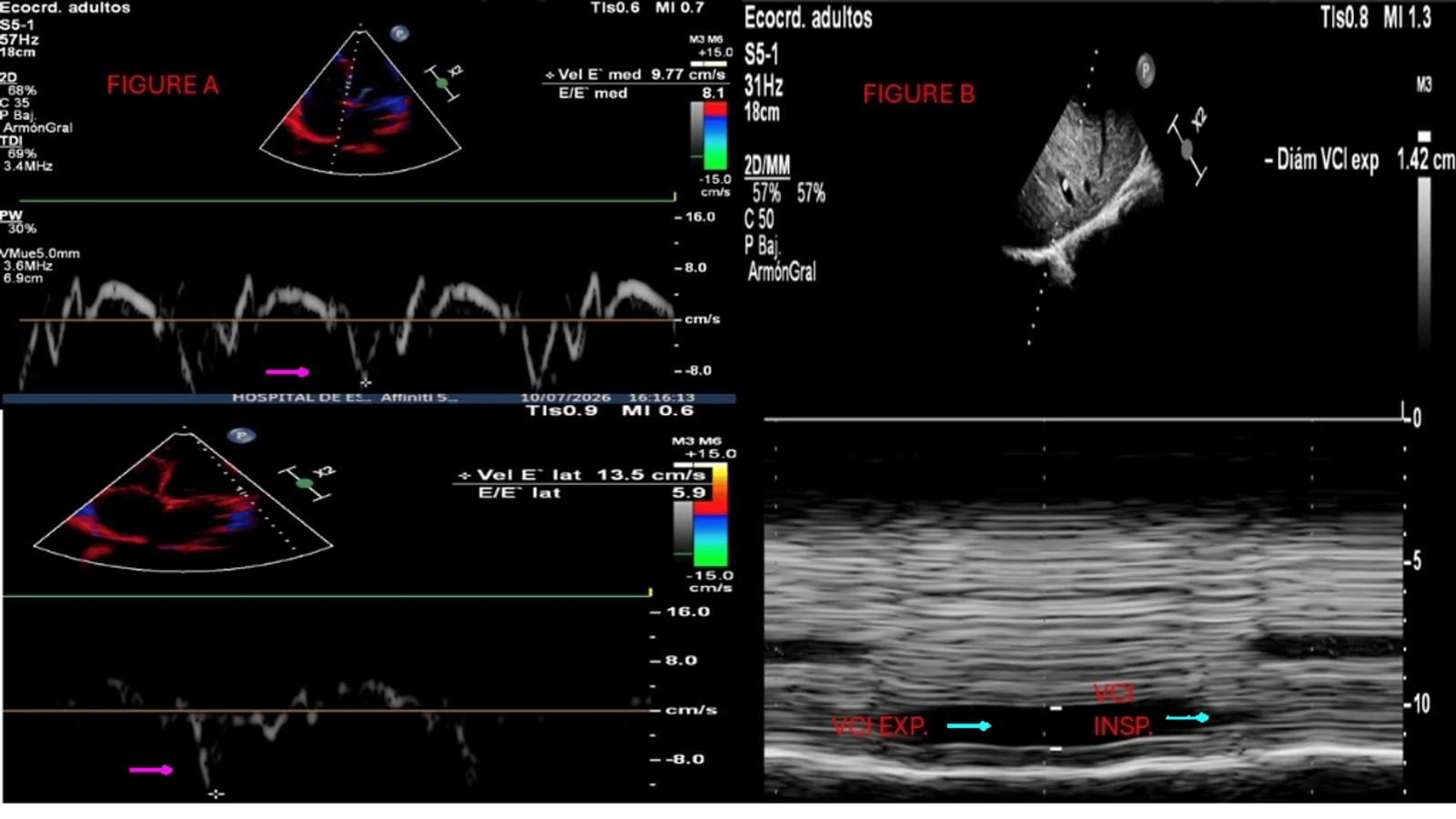
Functional echocardiographic resolution during outpatient follow-up (A) M-mode and tissue Doppler: The magenta arrows show resolution of the septal bounce and normalized annular velocities (lateral E' > septal E'). (B) Subcostal view: The cyan arrows indicate the diameter of the inferior vena cava during expiration (vena cava inferior (VCI) exp.) and its normalized >50% inspiratory collapse (VCI insp.).

Likewise, the follow-up transthoracic echocardiogram confirmed the resolution of the restrictive/constrictive physiology; the complete disappearance of the septal bounce and normalization of the respiratory variation of transmitral flow, which was less than 15%, were noted. Tissue Doppler analysis documented the restoration of the longitudinal mechanics of the ventricular free wall, recording an inversion of the previous pattern toward an ordinary physiological relationship where the lateral E' velocity was greater than the septal E' velocity. Finally, the subcostal view demonstrated normalization of the inferior vena cava diameter associated with the recovery of a dynamic inspiratory collapse greater than 50%, confirming the definitive decrease in filling pressures of the right cavities and the resolution of systemic venous hypertension (Figure 6). 

## Discussion

Constrictive pericarditis is a profound and progressive clinical syndrome characterized by a fibrotic, scarred, and often heavily calcified pericardium that functionally encapsulates the heart, severely impeding diastolic ventricular filling and precipitating a state of chronic, refractory systemic venous congestion [1-3]. CCP, historically designated as concretio cordis, represents the most extreme morphological manifestation of this disease continuum, signifying end-stage, irreversible structural transformation of the pericardial sac [1].

The etiological spectrum of constrictive pericarditis is broad; however, in contemporary practice in developed nations, it is most frequently encountered as a late complication of previous cardiac surgery, prior mediastinal radiation therapy, or idiopathic processes, while tuberculous pericarditis remains the predominant cause in developing regions [4,5]. The occurrence of concretio cordis specifically within the population of solid organ transplant recipients, and particularly among renal transplant patients, is exceptionally rare, with only a few isolated cases reported in the medical literature [5].

The pathophysiology of CCP in this unique demographic is complex and likely multifactorial. In the present case, the precise etiology is difficult to ascertain definitively. However, the patient's long-standing history of end-stage renal disease and prolonged pre-transplant hemodialysis strongly suggest a central role for chronic, indolent uremic pericarditis or recurrent, subclinical episodes of pericardial inflammation [6,7]. Furthermore, the chronic state of immunosuppression inherent to transplantation significantly increases vulnerability to opportunistic infections, which, even when subclinical or adequately treated, may trigger a slow, smoldering fibrotic cascade culminating in extensive pericardial calcification [5,8].

The clinical presentation of CCP is notoriously insidious and frequently misleading. Because left ventricular systolic function typically remains preserved until the late stages of the disease, the cardinal manifestations are predominantly those of severe, progressive right-sided heart failure: intractable peripheral edema, hepatic congestion, and marked ascites [1-3,9]. Consequently, as strikingly illustrated in this case, patients are frequently misdiagnosed for extended periods with chronic, end-stage liver disease (cardiac cirrhosis) or, in the specific context of renal transplantation, recurrent fluid overload secondary to subtle graft dysfunction [8,9]. This diagnostic latency often leads to prolonged, ineffective medical management with high-dose diuretics and repeated therapeutic paracenteses before the true cardiac etiology is recognized.

A high index of clinical suspicion is therefore absolutely essential when evaluating a transplant recipient presenting with unexplained, chronic systemic venous congestion, particularly in the presence of preserved LVEF and stable renal allograft function. The diagnostic algorithm must be prompt and multimodal. While the classical electrocardiographic finding of diffuse low voltage, as seen in this patient, is a hallmark of severe pericardial thickening or effusion, it lacks specificity [1]. The chest radiograph often provides the first critical diagnostic clue; the presence of an "armored heart" appearance, characterized by dense, contiguous rings of calcium encircling the cardiac silhouette, is practically pathognomonic for concretio cordis [8].

Advanced multimodal imaging is imperative to confirm the diagnosis, define the hemodynamics, and plan surgical intervention. Transthoracic echocardiography is the foundational tool, capable of identifying the thickened, hyper-reflective pericardium and, crucially, demonstrating the characteristic pathophysiological hallmarks of ventricular interdependence and restrictive physiology [1,5]. These include the exaggerated early diastolic interventricular septal shift ("septal bounce") and marked respiratory variations in mitral and tricuspid inflow velocities, definitively establishing the hemodynamic compromise [1,8].

These multimodal findings robustly fulfill the established Mayo Clinic criteria for constrictive pericarditis, which require the integration of elevated systemic venous pressures, characteristic echocardiographic hallmarks of enhanced ventricular interdependence and abnormal diastolic filling, and cross-sectional imaging confirmation of pericardial thickening. Specifically, our patient actively met these criteria: as clearly demonstrated in Figure 3, echocardiography revealed the hallmark "septal bounce," respiratory transvalvular variations, and hepatic vein flow reversal, while CT - serving alongside cardiac magnetic resonance as the gold-standard cross-sectional imaging modality - unequivocally confirmed the massive pericardial thickening and calcification (concretio cordis), collectively confirming the severe hemodynamic impairment [1,5,8].

Non-contrast CT of the chest is considered the gold standard for precisely delineating the distribution, thickness, and anatomical extent of the calcification, which is paramount for the surgical strategy [1,2]. CT exquisitely demonstrates the typical encasement of the right ventricle, diaphragmatic surface, and atrioventricular grooves, while also excluding significant associated pulmonary or mediastinal pathology [2,8].

The definitive and only effective treatment for symptomatic constrictive pericarditis is extensive surgical pericardiectomy [8,9]. This procedure is technically demanding and carries significant inherent risks, particularly in the presence of dense calcification (concretio cordis), which can deeply invade the epicardium, increasing the risk of major hemorrhage and catastrophic myocardial injury during dissection [8]. In the highly vulnerable population of renal transplant recipients, these surgical risks are profoundly amplified.

The traditional approach to complex pericardiectomy often involves the use of CPB to manage potential catastrophic bleeding and facilitate dissection in a decompressed heart [8]. However, the use of CPB in a patient with a solitary, functioning renal allograft introduces a constellation of severe, potentially fatal complications. CPB triggers a massive SIRS, induces significant coagulopathy, and generates prolonged periods of non-pulsatile flow and profound renal hypoperfusion [5,8]. This combination predictably precipitates AKI, significantly jeopardizing the long-term survival of the renal allograft and dramatically increasing overall perioperative mortality [5,8].

To mitigate these devastating risks, an off-pump surgical strategy is strongly advocated in this highly selected patient cohort [8,9]. As demonstrated in the present case, a meticulously executed subtotal off-pump pericardiectomy, aiming for anatomical clearance from phrenic nerve to phrenic nerve and comprehensive liberation of the diaphragmatic surface, can be performed safely and effectively without the support of CPB. This approach minimizes systemic inflammation, preserves physiological pulsatile perfusion, and is crucial for maximizing the protection of the renal allograft in this patient population [5,8].

When compared with previously published reports of renal transplant recipients undergoing pericardiectomy, our case shares clear similarities regarding the pre-transplant uremic history and the vulnerability of the renal allograft, but diverges remarkably due to the extreme morphological severity of massive pericardial calcification (concretio cordis) completely masking itself as advanced liver cirrhosis with refractory ascites. Furthermore, while historical protocols frequently depended on CPB, our successful execution of an off-pump surgical strategy highlights a vital technical distinction and reinforces safety, achieving complete hemodynamic decompression while fully preserving long-term solitary renal allograft function [5,8,9].

## Conclusions

CCP (concretio cordis) represents a rare and deceptive clinical entity in solid organ transplant recipients, where its insidious presentation can easily be misdiagnosed as advanced liver disease or graft dysfunction due to refractory ascites. This case emphasizes that a high index of clinical suspicion, coupled with prompt multimodal evaluation using echocardiography and CT, is essential for timely diagnostic recognition.

While surgical pericardiectomy remains the definitive treatment, managing such complex cases in the setting of a solitary renal allograft requires careful risk mitigation. As demonstrated by our patient's favorable outcome, a meticulously executed off-pump surgical strategy successfully achieved hemodynamic decompression and preserved renal allograft function, leading to symptom resolution, functional recovery, and the resumption of daily activities without limitations at mid-term follow-up. However, given the inherent limitations of a single-case observation, these findings cannot be generalized. While an off-pump approach offers a valuable, protective strategy for selected high-risk candidates, further studies are warranted to establish optimized management protocols for this vulnerable population.
